# ﻿Architecture of microtubule cytoskeleton in the hindgut cells of *Porcellioscaber*

**DOI:** 10.3897/zookeys.1225.116717

**Published:** 2025-02-05

**Authors:** Urban Bogataj, Polona Mrak, Jasna Štrus, Nada Žnidaršič

**Affiliations:** 1 University of Ljubljana, Biotechnical Faculty, Department of Biology, Večna pot 111, 1000 Ljubljana, Slovenia University of Ljubljana Ljubljana Slovenia

**Keywords:** Digestive system, hindgut, microtubules, morphogenesis, terrestrial isopods

## Abstract

The distribution and orientation of microtubules were investigated in cells of distinct shapes from different hindgut regions of adult *Porcellioscaber* Latreille, 1804 and during hindgut morphogenesis in late embryonic and early postembryonic development. All hindgut cells of adult *P.scaber* contain abundant apico-basal microtubules organized in extensive bundles, but the architecture of bundles is specific for distinct cells. In the anterior chamber the architecture of microtubule bundles closely coincides with different shapes of the cells in this hindgut region and are most prominent in hindgut cells associated with extensive muscles. The shape of cells that form the typhlosole and typhlosole channels is particularly complex. In the papillate region the microtubule bundles protrude between the infoldings of apical plasma membrane and the mitochondria are closely aligned along the microtubules, thus the microtubule bundles in the papillate region are likely involved in the stabilization of the apical labyrinth and positioning of mitochondria. During hindgut morphogenesis the apico-basal microtubule bundles are established relatively late, mainly during early postembryonic development. Morphogenesis of the typhlosole is characterized by coinciding changes in cell shape and microtubule arrangement.

## ﻿Introduction

Extensive apico-basally oriented bundles of microtubules are a hallmark of the hindgut epithelium of terrestrial isopods (Witkus et al. 1969; Bogataj et al. 2018). The apico-basally oriented arrangement of microtubules is a general characteristic of epithelial cells (Mays et al. 1994; Bartolini and Gundersen 2006), however this general arrangement is modified in different epithelia (Toya and Takeichi 2016).

### ﻿Arrangement of microtubules in epithelial cells and their reorganization during cell differentiation

Different epithelia in animal organisms provide a barrier between the external and internal environment. As such they are often exposed to mechanical strains, to which they cope using the interconnected system of cell junctions and cytoskeleton (Hagios et al. 1998). The rearrangement of this system is crucial also for the morphogenetic changes during the development of animal organisms (Schöck and Perrimon 2002). The microtubules are the stiffest among the main three cytoskeletal elements in eukaryotic cells and can form linear tracks that span the entire cells. Microtubules are polarized polymers and have complex assembly and disassembly dynamics. In non-polarized cells, the microtubules generally form radial arrays, with their minus ends positioned at the centrosome and their plus ends towards the cell periphery (Bornens 2002). The two best known functions of microtubules are that they form tracks for the directed intracellular transport and that they form the mitotic spindle during cell divisions for the segregation of chromosomes (Fletcher and Mullins 2010). Microtubules have also been proposed to influence cell shape and mechanics due to their abilities to resist the compressive forces (Brangwynne et al. 2006). During the differentiation of epithelial cells, the microtubules usually reorganize from the radial arrangement to the apico-basally polarized arrangement (Bré et al. 1987; Bacallao et al. 1989; Mimori-Kiyosue 2011). In polarized epithelial cells the majority of microtubules is apico-basally oriented, with their minus ends positioned in the apical cytosol and their plus ends in the basal cytosol. This arrangement is crucial for the establishment and maintenance of epithelial cell polarity and for the directed intracellular transport (Mays et al. 1994; Müsch 2004; Bartolini and Gundersen 2006).

The spatial arrangement of microtubules has been extensively investigated in mammalian epithelia, including intestinal cells, cochlear supporting cells of the inner ear and in various epithelial cells in culture. For arthropods, the most extensive data is available for the arrangement of microtubules in tracheal epithelial cells and pupal wing epithelial cells of *Drosophila* (Toya and Takeichi 2016; Muroyama and Lechler 2017). In other arthropods a lot of research has been conducted on extensive arrays of microtubules in tenocytes, which are specialized epidermal cells that serve as attachment sites of muscles to exoskeleton (Nakazawa et al. 1992; Tucker et al. 2004; Criel et al. 2005; Žnidaršič et al. 2012). Extensive arrays of microtubules have also been reported in various ectodermal epithelia with transportive functions, such as the gill epithelium of amphipods (Shires et al. 1994; Shires et al. 1995) and the hindgut epithelium of isopods (Witkus et al. 1969; Holdich and Ratcliffe 1970; Vernon et al. 1974; Wägele et al. 1981; Bogataj et al. 2018). Particularly in various mechanically burdened epithelial cells such as mammalian cochlear epithelial cells and tenocytes of arthropods the microtubules are often bundled and associated with different types of extensive cell junctions (Henderson et al. 1995; Tucker et al. 2004; Criel et al. 2005).

### ﻿Structure and function of the hindgut of terrestrial isopods

The digestive system of terrestrial isopods is composed of ectodermal foregut and hindgut that together form the entire alimentary canal and blind ending digestive glands which are connected to the stomach and represent the only endodermal part of the digestive system (Vernon et al. 1974; Hassall and Jennings 1975; Bettica et al. 1987; Storch and Štrus 1989; Štrus et al. 2008). Exception are some amphibious species where a narrow ring of endodermal midgut cells is present between the ectodermal stomach and the hindgut (Štrus et al. 1995).

The foregut consists of a tubular esophagus and stomach. Morphologically, the stomach represents the most complex part of the digestive system where the food is triturated and filtrated to the digestive glands by the movement of individual parts of the stomach, including different cuticular masticatory structures and filters, driven by the extensive musculature that surrounds the stomach (Storch 1987; Hames and Hopkin 1989; Storch and Štrus 1989). The main part of nutrient absorption takes place in the blind ending tubules of digestive glands, which receive filtered material from the stomach filters (Hames and Hopkin 1989).

The coarse solid food particles are directed to the hindgut which is the longest part of the digestive tract and consists of the anterior chamber, papillate region and rectum. The anterior chamber is characterized by a longitudinal dorsal fold termed typhlosole forming two parallel typhlosole channels on each side (Hassall and Jennings 1975; Hames and Hopkin 1989). In the anterior chamber the food is digested by the digestive enzymes and the liquid digestion products are transported along the typhlosole channels anteriorly, back to the stomach for further filtration and absorption in the digestive glands (Hames and Hopkin 1989). In the papillate region the dome-shaped basal parts of epithelial cells bulge into the haemocoel between the muscular net surrounding the hindgut, giving this part of the hindgut papillate appearance (Hassall and Jennings 1975; Hames and Hopkin 1989; Štrus et al. 1995). According to its structural characteristics the papillate region is considered a site of ions and water absorption (Vernon et al. 1974; Coruzzi et al. 1982; Palackal et al. 1984), important in terrestrial isopods to prevent excessive water loss. The rectum is separated from the papillate region by a muscular sphincter. Rectal epithelium is extensively folded and involved in the compaction of dry fecal pellets (Hames and Hopkin 1989; Storch and Štrus 1989).

The hindgut epithelium is monolayered and apically lined by a thin chitinous cuticle (Vernon et al. 1974; Palackal et al. 1984; Storch and Štrus 1989; Mrak et al. 2015). The hindgut cells have extensively infolded apical and basal plasma membrane and numerous mitochondria associated with the apical and basal infoldings, to support the active transport processes across the hindgut epithelium. The apical plasma membrane is connected to the hindgut cuticle with apical junctions that structurally resemble similar junctions in tenocytes. On lateral plasma membranes extensive and convoluted septate junctions are present, providing an efficient paracellular diffusion barrier (Vernon et al. 1974; Coruzzi et al. 1982; Storch and Štrus 1989; Bogataj et al. 2018).

### ﻿Microtubules in the hindgut cells of terrestrial isopods

Witkus et al. (1969) reported extensive apico-basally oriented bundles of microtubules concentrated in the lateral parts of hindgut epithelial cells in terrestrial isopod *Oniscusasellus* Linnaeus, 1758. They have proposed that these extensive bundles of microtubules are involved in several functions, including the structural support of extremely large hindgut cells. Similar apico-basally oriented bundles of microtubules in lateral parts of hindgut epithelial cells were later reported also in terrestrial isopods *Armadillidiumvulgare* Latreille, 1804 and *Porcellioscaber* Latreille, 1804 and in marine isopods *Dynamenebidentata* Adams, 1800 and *Cyathuracarinata* Krøyer, 1847 (Holdich and Ratcliffe 1970; Vernon et al. 1974; Wägele et al. 1981; Bogataj et al. 2018). Authors of studies performed on *A.vulgare* and *P.scaber* report that the bundles of microtubules in the hindgut cells associate with apical and basal plasma membrane infoldings (Vernon et al. 1974) and with junctions between the apical plasma membrane and cuticle (Bogataj et al. 2018).

### ﻿Hindgut morphogenesis during development of *Porcellioscaber*

The embryonic and early postembryonic development of *Porcellioscaber* and other terrestrial isopods takes place in an osmotically regulated aqueous environment of marsupium, which is a fluid filled structure at the ventral side of a gravid female body (Surbida and Wright 2001). The eggs and developing embryos are enclosed in two embryonic envelopes: the outer chorion and the inner vitelline membrane (Wolff 2009; Milatovič et al. 2010; Mrak et al. 2012). According to Milatovič et al. (2010), the embryonic development of *P.scaber* lasts approximately 25 days and is divided into 20 developmental stages. Stages S1–S5 comprise the early-stage embryos which contain large amount of yolk and have no visible limb buds. Stages S6–S15 comprise the mid-stage embryos which are characterized by the appearance of the limb buds and are dorsally bent. During mid-embryogenesis, the yolk is gradually enclosed into the developing digestive glands and the formation of foregut and hindgut begins with the invagination of proctodeum and stomodeum. The proctodeum appears in stage S6 embryos and gradually elongates towards the stomodeum during stages S6–S14. Stomodeum and proctodeum fuse into a continuous digestive tube at the end of mid-embryogenesis in stage S15 embryos. Stages S16–S19 comprise late-stage embryos. Stage S16 embryos hatch from the chorion and become ventrally bent. In late-stage embryos the cuticular structures in the filtering regions of the stomach are formed and in S18 embryos the division of the hindgut into the anterior chamber and the papillate region becomes apparent (Štrus et al. 2008; Wolff 2009; Milatovič et al. 2010). Stage S20 designates the marsupial manca after the release from the vitelline membrane (Milatovič et al. 2010).

The term manca denotes all juvenile animals that already closely resemble adult animals but still lack the 7^th^ pair of pereopods. The marsupial mancae develop within the marsupium for approximately ten more days. Mrak et al. (2012) defined early-stage, mid-stage, and late-stage marsupial mancae according to differences in size, pigmentation, motility, and amount of yolk in the digestive glands. Marsupial mancae already have distinct filters, ridges, and folds in the stomach. The typhlosole in the anterior chamber is elaborated and functional and the hindgut cuticle consists of procuticle and thin epicuticle (Štrus et al. 2008; Mrak et al. 2015; Štrus et al. 2019). After the release from the marsupium, the mancae develop as postmarsupial mancae for up to additional 20 days before reaching the juvenile stage with seven pairs of pereopods (Tomescu and Craciun 1987). During the postmarsupial development certain ultrastructural features of hindgut cells, such as the apical and basal labyrinths and the septate junctions, are further elaborated (Bogataj et al. 2019).

### ﻿Aim

The outstanding ultrastructural feature of isopod hindgut cells are extensive apico-basal bundles of microtubules. In order to better understand the relation between the architecture of microtubule bundles and the shape and function of cells we have investigated for the first time the arrangement of microtubules in cells from different hindgut regions of adult *P.scaber* animals and in hindgut cells of selected developmental stages. For this, we have employed a combination of immunofluorescent labelling of α-tubulin on paraffin and semithin plastic sections and the ultrastructural analysis with transmission electron microscopy. The aim of the current study is primarily to provide a detailed description of microtubule organization in morphologically and functionally different hindgut regions of *P.scaber* and to discuss the obtained results in respect to the functions that the analyzed hindgut regions have been assigned to. Furthermore, we aim to establish how the microtubule arrays in the hindgut form during the embryonic and early postembryonic development.

## ﻿Materials and methods

### ﻿Specimens of *Porcellioscaber*

Adult animals as well as embryos and mancae of *Porcellioscaber* were obtained from a laboratory culture that had been maintained in a glass terrarium with ground cover of soil and leaf litter. Animals were bred at 25 °C, high humidity, and 12 h light/12 h dark cycle. Embryos and marsupial mancae were isolated from the marsupia of gravid females. The developmental stages of embryos were determined morphologically according to Milatovič et al. (2010) and the developmental stages of marsupial mancae according to Mrak et al. (2012). Investigated were the late embryos in stage S18, early-stage marsupial mancae and late-stage marsupial mancae. Postmarsupial mancae have been sampled at the day of the release and 1 week after the release from the marsupium. Gravid females were held separately in plastic petri dishes that each contained a moist filter paper and a dry leaf until mancae release.

### ﻿Fixation and embedding procedures of samples for the immunohistochemistry

To obtain histological paraffin sections of adult animals, the animals were first anesthetized in a petri dish containing a small piece of cotton wool soaked in diethyl ether. Some of the anesthetized animals were transversely cut in half and both halves were processed whole. Some of the anesthetized animals were dissected, and the isolated hindguts were processed. The embryos, marsupial and postmarsupial mancae were processed whole. All samples were fixed in 4% formaldehyde in 0.1 mol/L HEPES buffer (pH 7.2). After the fixation the samples were rinsed with 0.1 mol/L HEPES buffer. The samples were then dehydrated in graded series of ethanol (50%, 70%, 80%, 90%, 96% and 100% ethanol) and cleared in xylene. After the dehydration and clearing the samples were infiltrated with Paraplast at 60 °C (2 changes overnight) and embedded in Paraplast on embedding station HistoCore Arcadia (Leica). Seven µm thick paraffin sections were prepared on a Leica RM 2265 microtome.

To obtain semithin sections of LR-White embedded samples, the hindguts were isolated from the anesthetized adult animals, while the embryos and mancae were processed whole. The samples were fixed either in 0.25% glutaraldehyde and 2% formaldehyde in 0.1 mol/L HEPES buffer (pH 7.2) or in 4% formaldehyde in 0.1 mol/L HEPES buffer (pH 7.2). After the fixation, the samples were rinsed in 0.1 mol/L HEPES buffer and dehydrated in graded series of ethanol (30%, 50%, 70%, 90% and 100% ethanol). Dehydrated samples were infiltrated with acrylic resin LR-White (London Resin Company Ltd). The resin was thermally cured in gelatin capsules at 60 °C for 24 h. 0.5 µm thick semithin sections were prepared using glass knives or a diamond histo knife (Diatome) on an ultramicrotome Reichert Ultracut S (Leica). Semithin sections are considerably thinner than the paraffin sections and thus higher resolution images of fluorescently labelled microtubules can be obtained. Some semithin sections were stained with Azure II – Methylene Blue for histological examinations, while most of the sections were used for immunolabelling.

### ﻿Immunofluorescent labelling of microtubules on paraffin and LR-White sections

To visualize the microtubules immunolabelling of α-tubulin was performed. The α-tubulin forms dimers with β-tubulin which assemble to form the microtubules. Before the immunolabelling the paraffin sections were deparaffinized in xylene and rehydrated in graded series of alcohol (100% propanol, 96% ethanol, 70% ethanol) and distilled water. The rehydrated paraffin sections and LR-White sections were rinsed in PBS (phosphate buffered saline), blocked with 1% BSA (bovine serum albumin) in PBS and incubated overnight at 4 °C with primary mouse antibodies monoclonal anti-α-tubulin (mouse IgG1 isotype) (Sigma-Aldrich, catalog no.: T9026) diluted 1:500 in 1% BSA in PBS. After the incubation with primary antibodies the sections were rinsed with PBS and incubated with secondary goat antibodies against mouse IgG conjugated with fluorescent dye AlexaFluor 488 (ThermoFisher Scientific, A-11001) and diluted 1:300. Following the incubation with secondary antibodies sections were rinsed with PBS and covered with mounting medium FluoroShield with DAPI (4′,6-Diamidine-2′-phenylindole dihydrochloride) (Sigma-Aldrich) to counterstain cell nuclei.

Immunolabelled sections were examined with AxioImager Z.1 microscope (Zeiss) using the fluorescence and differential interference contrast (DIC) imaging modes. For the fluorescence microscopy filter sets appropriate for DAPI (excitation BP 365/12, beam splitter FT 395, emission LP 397) and AlexaFluor 488 (excitation BP 450–490, beam splitter FT 510, emission LP 515) were used. The digital micrographs were acquired with HRc AxioCam camera (Zeiss) using the AxioVision software (Zeiss). The images were processed (brightness and contrast adjusted) and the overlays of epifluorescence and DIC images were prepared in FIJI (ImageJ) software.

### ﻿Ultrastructural analysis of resin embedded samples with transmission electron microscopy

The ultrastructural information on the arrangement of microtubules in hindgut cells was obtained by transmission electron microscopy of ultrathin sections of resin embedded samples. The adult animals were dissected to isolate the hindguts and the embryos and mancae were processed whole. The samples were chemically fixed in 2.5% glutaraldehyde and 4% paraformaldehyde in 0.1 mol/L HEPES buffer. After the fixation the samples were rinsed with 0.1 mol/L HEPES buffer and postfixed in 1% OsO_4_ in 0.1 mol/L HEPES buffer. Then the samples were dehydrated in graded series of ethanol and acetone (50%, 70%, 90%, 100% ethanol, and 100% acetone) and infiltrated with epoxy resin Agar 100 (AgarScientific). The resin was polymerized in silicone molds at 60 °C for 24 h. The semithin sections were prepared as described for LR-White embedded specimens. 70 nm thick ultrathin sections were cut with a diamond ultra knife (Diatome) on the ultramicrotome Reichert Ultracut S (Leica), transferred to copper mesh grids and contrasted with 1% uranyl acetate and 0.1% lead citrate. Prepared ultrathin sections were examined with a Phillips CM100 transmission electron microscope and the electron micrographs acquired with a Bioscan 792 (Gatan) and an Orius SC200 (Gatan) digital camera, using the Digital Micrograph (Gatan) software.

## ﻿Results

### ﻿Histological structure of the typhlosole and the shape of hindgut cells in adult *P.scaber*

**Figure 1.**
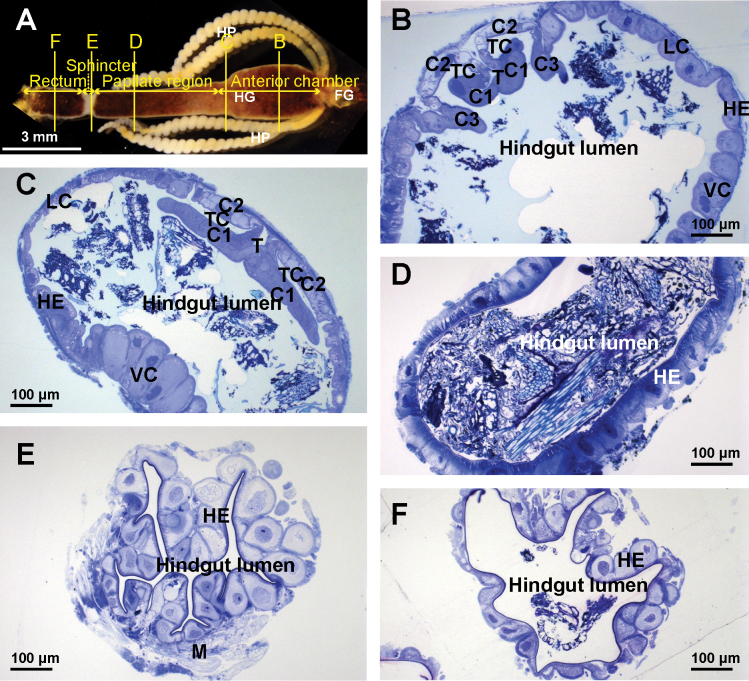
Anatomical and histological structure of the hindgut in adult *P.scaber***A** anatomical structure and hindgut regions. Lines B, C, D, E, and F denote where the cross-sections depicted in images B, C, D, E and F were made **B** cross-section across the anterior part of the anterior chamber **C** cross-section across the posterior part of the anterior chamber **D** cross-section across the papillate region **E** cross-section across sphincter **F** cross-section across rectum. Abbreviations: C1 – curved cells forming the typhlosole; C2 – wide cells enclosing the typhlosole channels; C3 – curved cells forming ridges on each side of the typhlosole; FG – foregut; HE – hindgut epithelium; HG – hindgut; HP – hepatopancreas; LC – lateral cells; M – muscles; T – typhlosole; TC – typhlosole channels; VC – ventral cells.

In the adult hindgut four regions can be distinguished at the level of gross anatomy: anterior chamber, papillate region, sphincter region and rectum (Fig. 1A). The anterior chamber in its anterior part is characterized by a relatively narrow typhlosole and two deep typhlosole channels (Fig. 1B). The dorsal cells that constitute the typhlosole and two typhlosole channels have very complex shapes. Typhlosole cells are highly curved in cross-section. The two cells that enclose the typhlosole channels immediately on each side of the typhlosole are wide and flattened and the two cells further away are curved in cross-section and form two ridges protruding into the hindgut lumen on each side of the typhlosole. The remaining hindgut cells in the anterior chamber, which build the lateral and ventral hindgut wall, are isodiametric to prismatic with dome shaped apical parts protruding into the hindgut lumen. In the posterior part of the anterior chamber the typhlosole widens considerably and becomes anvil shaped, and the two typhlosole channels are very narrow (Fig. 1C). In cross-section, the curvature of the typhlosole cells is even more pronounced than anteriorly. The cells enclosing the typhlosole channels are wider and the two ridges on each side of the typhlosole are absent. Cells that build the lateral hindgut wall are relatively small and isodiametric. The ventral cells are very large, prismatic and with apical parts protruding deep into the hindgut lumen. In the papillate region all the cells of the hindgut epithelium look similar. They are isodiametric with dome shape basal parts protruding into the haemocoel (Fig. 1D). In the sphincter region extensive circular muscles surround the hindgut epithelium, which forms deep folds that narrow the hindgut lumen. The hindgut cells are isodiametric with dome shaped basal parts protruding into the haemocoel (Fig. 1E). In the rectum the hindgut lumen becomes wide again and the musculature surrounding the hindgut is less extensive than in the sphincter region. The shape of the hindgut cells is the same as in the sphincter region (Fig. 1F).

### ﻿Specific arrangement of microtubules in the epithelial cells in distinct hindgut regions of adult *P.scaber*

**Figure 2.**
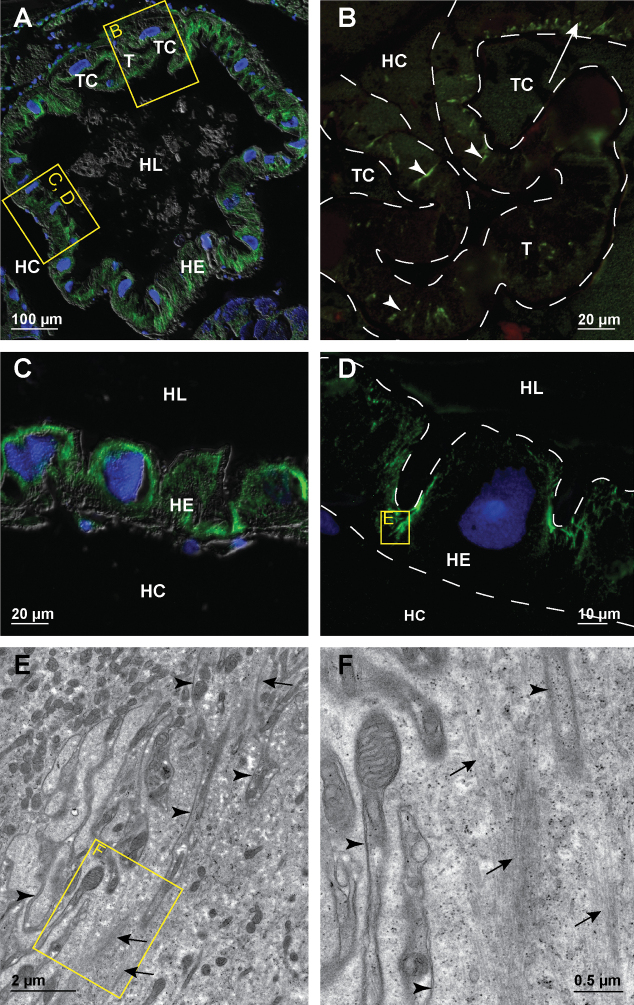
Microtubules in hindgut cells in the anterior chamber of adult *P.scaber***A** overlay of epifluorescence and DIC images obtained on paraffin section showing microtubules in green (AlexaFluor 488) and cell nuclei in blue (DAPI). Image depicts cross-section across the anterior chamber **B** image of immunofluorescent-labelled microtubules in green on LR-White section. Image depicts equivalent area as denoted by frame B in image A. Arrowheads point to transcellular microtubule bundles in typhlosole cells oriented perpendicular to the apical cell surface. Arrow points to short profiles of microtubule bundles present in the apical cytosol of wide cells around the typhlosole channels. The white dashed lines outline the apical and basal surface of epithelial cells **C** overlay of epifluorescence and DIC images obtained on paraffin section showing microtubules in green (AlexaFluor 488) and cell nuclei in blue (DAPI). Image depicts equivalent area as denoted by the frame C, D in image A **D** overlay of epifluorescence images obtained on semithin LR-White section showing microtubules in green (AlexaFluor 488) and cell nuclei in blue (DAPI). Image depicts equivalent area as denoted by the frame C, D in image A. The white dashed lines outline the apical and basal surface of hindgut epithelium **E** electron micrograph showing equivalent area as denoted by frame E in image D. Arrows point to abundant microtubule bundles near the lateral plasma membrane (arrowheads) **F** higher magnification of an area denoted by frame F in image E. Arrows point to bundled individual microtubules near the lateral plasma membrane (arrowheads). Abbreviations: HC – hemocoel; HE – hindgut epithelium; HL – hindgut lumen; T – typhlosole; TC – typhlosole channels.

We compared the abundance and arrangement of microtubules in hindgut cells of diverse shapes and sizes within the anterior chamber, papillate region, sphincter region and rectum. In the anterior chamber the epithelial cells contain extensive arrays of microtubules (Fig. 2A). The cells of the typhlosole contain long transcellular bundles oriented perpendicular to the apical cell surface. The hindgut cells around the two typhlosole channels contain short profiles of densely stacked microtubule bundles present only in the apical cytosol (Fig. 2B). The remaining epithelial cells that form the lateral and ventral hindgut walls contain thick bundles of microtubules concentrated in the lateral cytosol (Fig. 2C, D). The ultrastructural analysis of hindgut epithelial cells in the anterior chamber reveals extensive apico-basal bundles of microtubules associated with lateral borders of cells. These bundles completely fill large areas of lateral cytosol (Fig. 2E, F).

**Figure 3.**
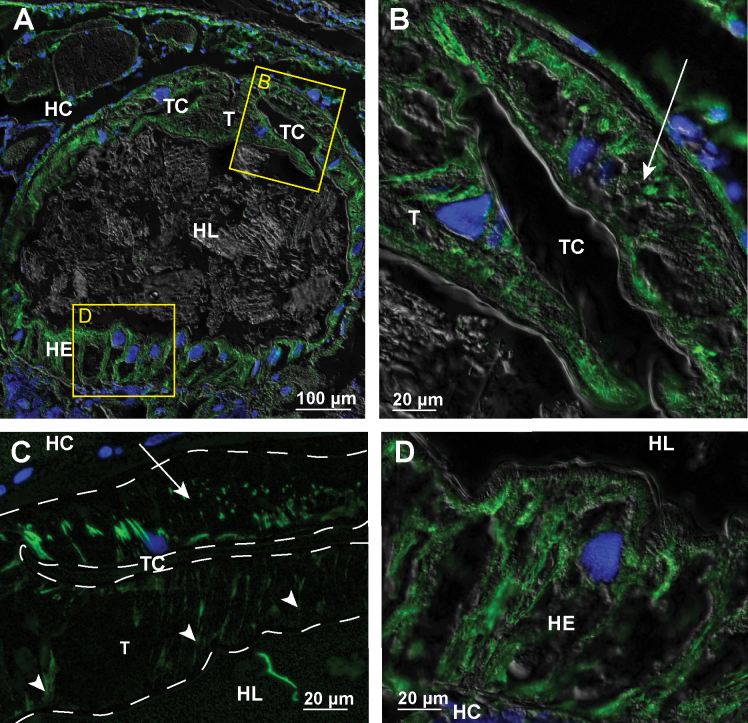
Microtubules in hindgut cells in the posterior part of anterior chamber of adult *P.scaber***A** overlay of epifluorescence and DIC images obtained on paraffin section showing microtubules in green (AlexaFluor 488) and cell nuclei in blue (DAPI). Image depicts cross-section across the anterior chamber near the transition to the papillate region **B** overlay of epifluorescence and DIC images obtained on paraffin section showing microtubules in green (AlexaFluor 488) and cell nuclei in blue (DAPI). Image depicts area denoted by the frame B in image A **C** overlay of epifluorescence images obtained on semithin LR-White section showing microtubules in green (AlexaFluor 488) and cell nuclei in blue (DAPI). Image depicts equivalent area as image B. Arrow points to large cell that surround the typhlosole channels and contain abundant microtubule bundles. Arrowheads point to parallel bundles of microtubules oriented perpendicular to the apical surface of typhlosole cells. The white dashed lines outline the contour of hindgut epithelium **D** overlay of epifluorescence and DIC images obtained on paraffin section showing microtubules in green (AlexaFluor 488) and cell nuclei in blue (DAPI). Image depicts area denoted by the frame D in image A. Abbreviations: HC – hemocoel; HE – hindgut epithelium; HL – hindgut lumen; T – typhlosole; TC – typhlosole channels.

In the posterior part of the anterior chamber close to the papillate region the apico-basally oriented bundles of microtubules are particularly extensive (Fig. 3A). The most prominent transcellular microtubular bundles are present in dorsal hindgut cells of the typhlosole and typhlosole channels (Fig. 3B, C) and in high dome shaped ventral cells (Fig. 3D). In cells around the typhlosole channels the microtubules occur near the apical and the basal surface of these cells (Fig. 3B). In the dorsal epithelium, both the typhlosole cells and the wide cells around the typhlosole channels contain parallel bundles of microtubules (Fig. 3C). In highly folded typhlosole cells, the microtubule bundles are oriented perpendicular to the apical cell surface.

**Figure 4.**
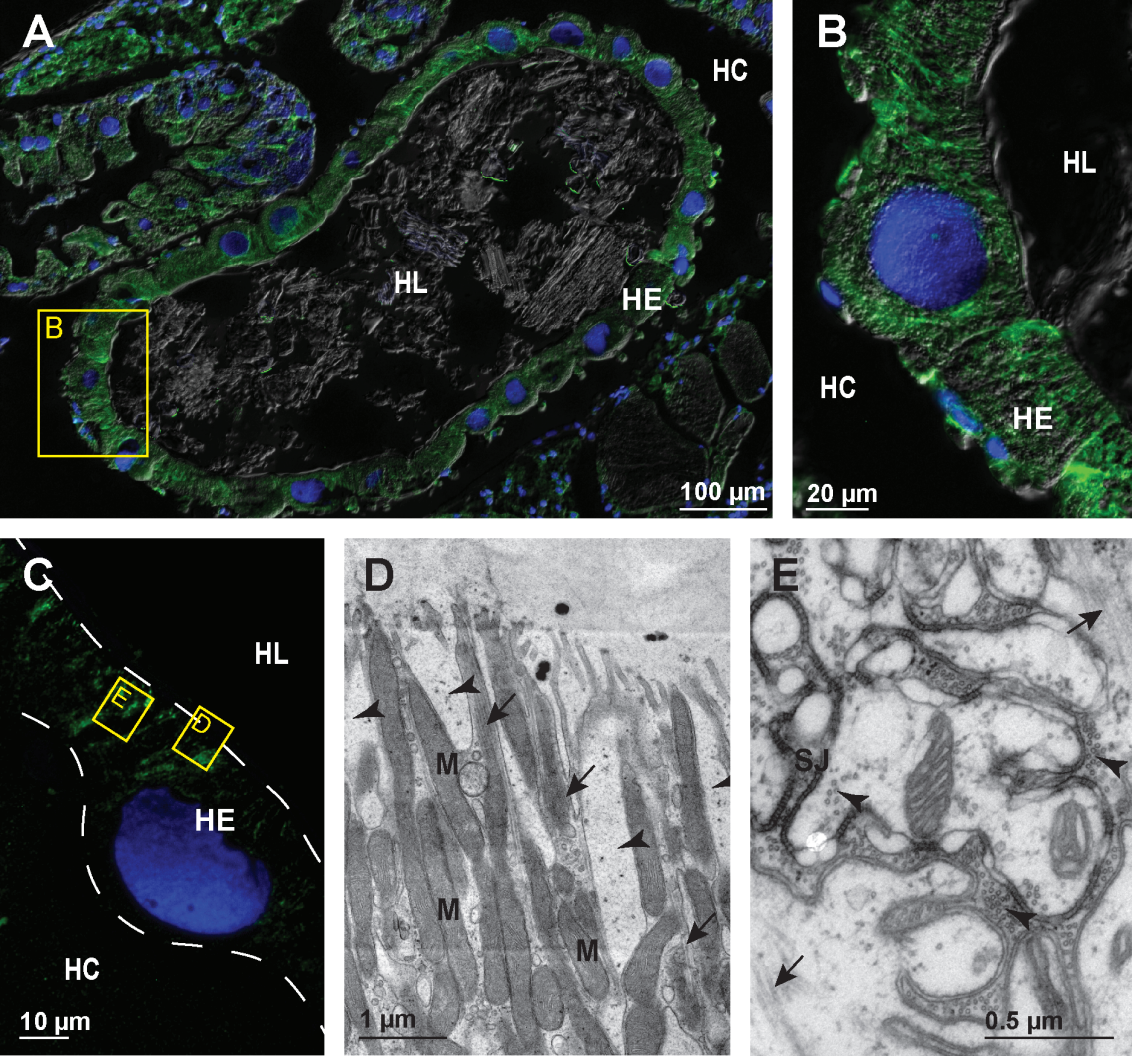
Microtubules in hindgut cells in the papillate region of adult *P.scaber***A** overlay of epifluorescence and DIC images obtained on paraffin section showing microtubules in green (AlexaFluor 488) and cell nuclei in blue (DAPI). Image depicts cross-section across the papillate region **B** overlay of epifluorescence and DIC images obtained on paraffin sections showing microtubules in green (AlexaFluor 488) and cell nuclei in blue (DAPI). Image depicts area denoted by the frame B in image A **C** overlay of epifluorescence images obtained on semithin LR-White section showing microtubules in green (AlexaFluor 488) and cell nuclei in blue (DAPI). Image depicts equivalent area as image B. The white dashed lines in figure C outline the apical and basal surface of hindgut epithelium **D** electron micrograph showing equivalent area as denoted by frame D in image C. Arrows point to microtubule bundles in the cytosol between the infoldings of apical plasma membrane which are indicated by the arrowheads **E** electron micrograph showing equivalent area as denoted by frame E in image C. Arrowheads point to numerous cross-sectioned microtubules in the vicinity of septate junctions. Arrows point to longitudinally sectioned apico-basal microtubules. Abbreviations: HC – hemocoel; HE – hindgut epithelium; HL – hindgut lumen; M – mitochondria; SJ – septate junctions.

In the papillate region all cells in the hindgut epithelium contain fine apico-basally oriented bundles of microtubules (Fig. 4A, B). This arrangement of microtubules is general throughout the papillate region epithelium. The bundles of microtubules appear thinner than in the anterior chamber and are evenly distributed throughout the cytosol (Fig. 4C). At the ultrastructural level prominent bundles of microtubules are evident along the narrow cytoplasmic bands between the infoldings of apical plasma membrane (Fig. 4D). These microtubules are also in close contact with numerous mitochondria present in the apical cytosol. Cross-sectioned profiles of microtubules oriented perpendicular to the apico-basal axis of epithelial cells are often observed in the vicinity of septate junctions in the papillate region (Fig. 4E).

**Figure 5.**
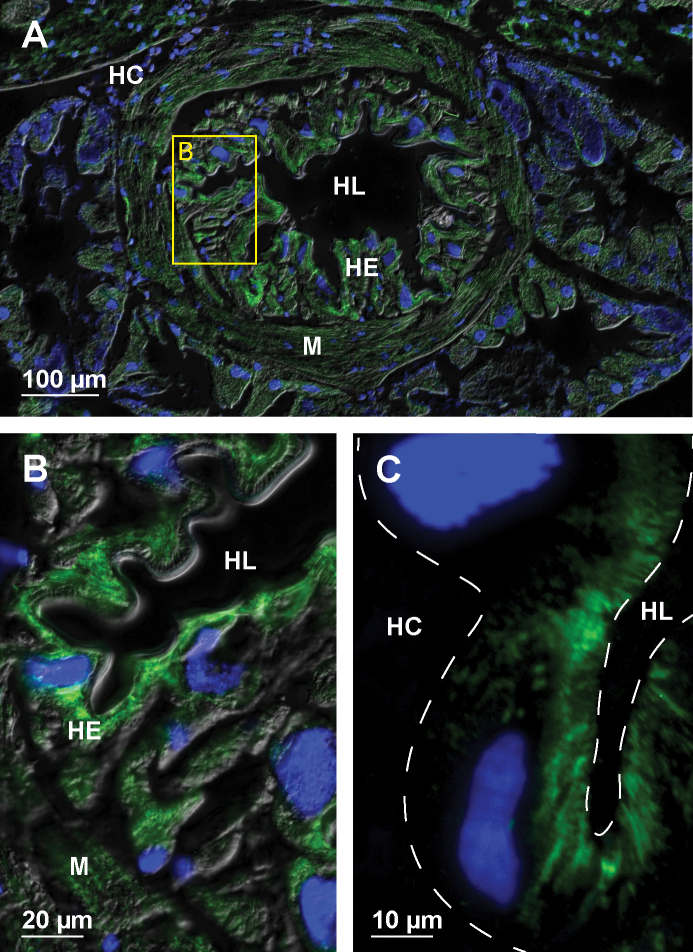
Microtubules in hindgut cells in the sphincter region in adult *P.scaber***A** overlay of epifluorescence and DIC images obtained on paraffin section showing microtubules in green (AlexaFluor 488) and cell nuclei in blue (DAPI). Image depicts cross-section across the sphincter region **B** overlay of epifluorescence and DIC images obtained on paraffin section showing microtubules in green (AlexaFluor 488) and cell nuclei in blue (DAPI). Image depicts area denoted by frame B in image A **C** overlay of epifluorescence images obtained on semithin LR-White section showing microtubules in green (AlexaFluor 488) and cell nuclei in blue (DAPI). Image depicts cross-section of hindgut epithelium in sphincter region at high magnification. The white dashed lines in figure C outline the contour of hindgut epithelium. Abbreviations: HC – hemocoel; HE – hindgut epithelium; HL – hindgut lumen; M – muscles.

In the sphincter region between the papillate region and the rectum the hindgut epithelium is relatively thin (Fig. 5A), and the hindgut cells contain numerous microtubules concentrated in the apical cytosol (Fig. 5B). Fine bundles of microtubules are oriented in the apico-basal direction and limited to the apical cytosol of cells as discernible on semi-thin LR-White sections (Fig. 5C).

**Figure 6.**
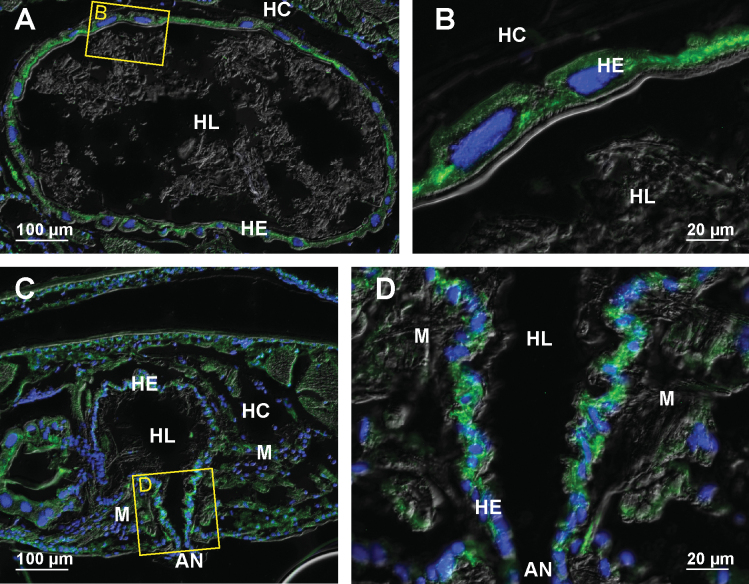
Microtubules in hindgut cells in the rectum in adult *P.scaber***A** overlay of epifluorescence and DIC images obtained on paraffin section showing microtubules in green (AlexaFluor 488) and cell nuclei in blue (DAPI). Image depicts cross-section across the rectum **B** overlay of epifluorescence and DIC images obtained on paraffin section showing microtubules in green (AlexaFluor 488) and cell nuclei in blue (DAPI). Image depicts area denoted by frame B in image A **C** overlay of epifluorescence and DIC images obtained on paraffin section showing microtubules in green (AlexaFluor 488) and cell nuclei in blue (DAPI). Image depicts cross-section across the rectum near the anal opening **D** overlay of epifluorescence and DIC images obtained on paraffin section showing microtubules in green (AlexaFluor 488) and cell nuclei in blue (DAPI). Image depicts area denoted by frame D in image C. Abbreviations: AN – anal opening; HC – hemocoel; HE – hindgut epithelium; HL – hindgut lumen; M – dilator muscles.

The hindgut epithelium is also relatively thin in the rectum (Fig. 6A). The arrangement of microtubule bundles in epithelial cells of the rectum is similar as in the sphincter region, with microtubules concentrated in the apical cytosol (Fig. 6B). In the posterior part of the rectum toward the anal opening, strong dilator muscles are attached to the hindgut epithelium (Fig. 6C) in contrast to the remaining part of the rectum. In this posterior part the epithelial cells are relatively small and almost filled with microtubules (Fig. 6D).

### ﻿Arrangement of microtubules in the hindgut cells of embryos and mancae

**Figure 7.**
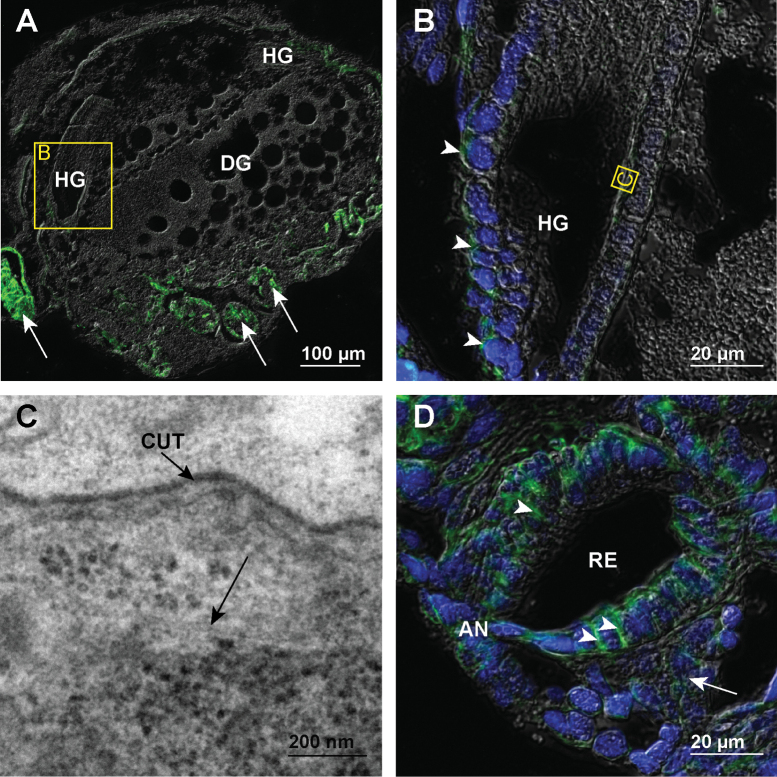
Microtubules in hindgut cells of late embryos of stage S18 **A** overlay of epifluorescence and DIC images obtained on paraffin section showing microtubules in green (AlexaFluor 488). Image depicts longitudinal section of the embryo. Arrows point to distinct fluorescent labelling of microtubules in epidermis of the limb buds **B** overlay of epifluorescence and DIC images obtained on paraffin section showing microtubules in green (AlexaFluor 488) and cell nuclei in blue (DAPI). Image depicts area denoted by frame B in image A. Arrowheads point to diffuse fluorescent labelling in the hindgut **C** electron micrograph of equivalent area as denoted by frame C in image B. Arrow points to an individual microtubule in the apical cytosol **D** overlay of epifluorescence and DIC images obtained on paraffin section showing microtubules in green (AlexaFluor 488) and cell nuclei in blue (DAPI). Image depicts cross-section of the rectum near the anal opening. Arrowheads point to apico-basal bundles of microtubules in the rectum. An arrow points to distinct dilator muscles surrounding the rectum. Abbreviations: AN – anal opening; CUT – cuticle; DG – digestive glands; HG – hindgut; RE – rectum.

In the paraffin sections of the whole late embryos of stage S18 the immunofluorescent labelling of α-tubulin shows only a weak reaction in the hindgut cells. A strong positive reaction is discernible in the epidermis of the limb buds (Fig. 7A). In the hindgut epithelium along most of the alimentary canal the reaction of fluorescent labelling is weak and diffusely distributed in the cytosol (Fig. 7B). At the ultrastructural level we have observed individual microtubules in the cytosol of hindgut cells (Fig. 7C). The distinct apico-basally oriented bundles are discernible only in the most posterior part of the alimentary canal, i.e., the rectal epithelium (Fig. 7D).

**Figure 8.**
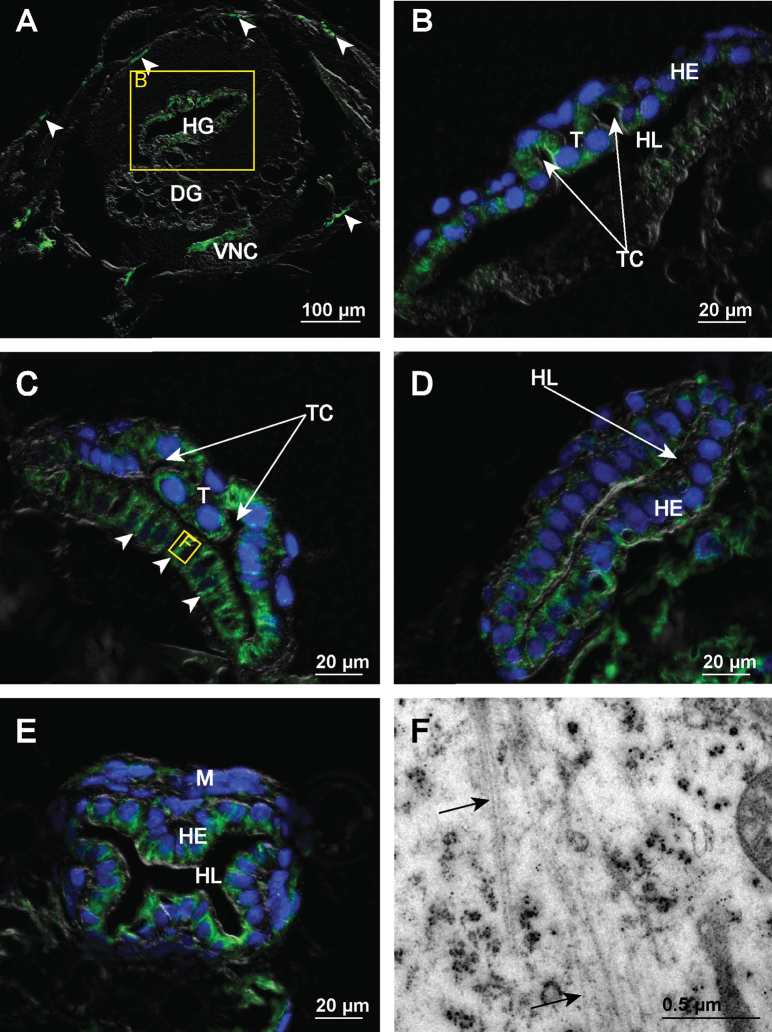
Microtubules in hindgut cells of early-stage marsupial mancae **A** overlay of epifluorescence and DIC images obtained on paraffin section showing microtubules in green (AlexaFluor 488). Image depicts a transverse section across the anterior part of manca. Arrowheads point to microtubules in dorsal tergites **B** overlay of epifluorescence and DIC images obtained on paraffin section showing microtubules in green (AlexaFluor 488) and cell nuclei in blue (DAPI). Image depicts area denoted by frame B in image A **C** overlay of epifluorescence and DIC images obtained on paraffin section showing microtubules in green (AlexaFluor 488) and cell nuclei in blue (DAPI). Image depicts hindgut epithelium at the transition between the anterior chamber and the papillate region. Arrowheads point to cells containing distinct apico-basal bundles of microtubules **D** overlay of epifluorescence and DIC images obtained on paraffin section showing microtubules in green (AlexaFluor 488) and cell nuclei in blue (DAPI). Image depicts hindgut epithelium from the anterior part of the papillate region **E** overlay of epifluorescence and DIC images obtained on paraffin section showing microtubules in green (AlexaFluor 488) and cell nuclei in blue (DAPI). Image depicts hindgut epithelium from the posterior part of the papillate region **F** electron micrograph of equivalent area as denoted by frame F in image C showing the cell in the ventral hindgut epithelium. Arrows point to small bundles of microtubules in the cytosol. Abbreviations: DG – digestive glands; HE – hindgut epithelium; HG – hindgut; HL – hindgut lumen; M – muscles; T – typhlosole; TC – typhlosole channels; VNC – ventral nerve cord.

In marsupial mancae the immunofluorescent labelling of microtubules is considerably more pronounced than in the late embryos, on both paraffin and semithin sections. We did not observe any considerable differences in labelling between the early-stage and late-stage marsupial mancae. The most pronounced fluorescent labelling is detectable in certain sites of tergite’s epidermis, presumably tenocytes, in the hindgut epithelium and in the cells of the ventral nerve cord (Fig. 8A). In the hindgut epithelium the apico-basal orientation of microtubule bundles is evident in all observed regions of the hindgut. In the anterior chamber there is a distinct difference in the abundance of microtubules between the dorsal part and the ventral part of the hindgut epithelium. The labelling of microtubules is very distinct in the dorsal epithelium, particularly in cells of typhlosole and typhlosole channels. Cells in the ventral epithelium are only weakly labelled (Fig. 8B). The bundles of microtubules are particularly extensive in ventral and dorsal cells at the transition between the anterior chamber and the papillate region (Fig. 8C). In the papillate region the distribution of the microtubules in epithelial cells differs between anterior part and posterior part of the region. In the anterior part fine bundles of microtubules span the entire cells in the apico-basal direction (Fig. 8D), while in the posterior part the bundles are condensed in the apical cytosol (Fig. 8E). At the ultrastructural level it is evident that microtubules associate laterally with one another and form small apico-basally oriented bundles (Fig. 8F). In the terminal part of the rectum near the anal opening the arrangement of apico-basal bundles of microtubules is similar as in the late embryos of stage S18.

**Figure 9.**
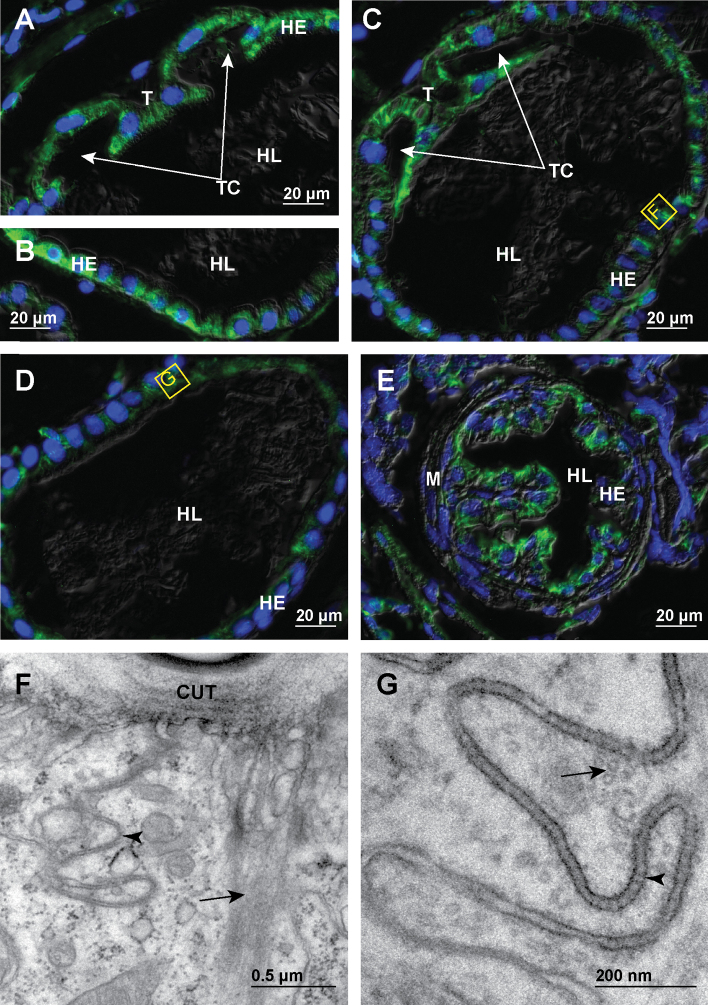
Microtubules in hindgut cells of postmarsupial mancae 1 week after their release from the marsupium **A** overlay of epifluorescence and DIC images obtained on paraffin section showing microtubules in green (AlexaFluor 488) and cell nuclei in blue (DAPI). Image depicts dorsal hindgut epithelium in the anterior chamber **B** overlay of epifluorescence and DIC images obtained on paraffin section showing microtubules in green (AlexaFluor 488) and cell nuclei in blue (DAPI). Image depicts hindgut epithelium of the lateral hindgut wall in the anterior chamber **C** overlay of epifluorescence and DIC images obtained on paraffin section showing microtubules in green (AlexaFluor 488) and cell nuclei in blue (DAPI). Image depicts hindgut epithelium at the transition between the anterior chamber and the papillate region **D** overlay of epifluorescence and DIC images obtained on paraffin section showing microtubules in green (AlexaFluor 488) and cell nuclei in blue (DAPI). Image depicts hindgut epithelium in the anterior part of the papillate region **E** overlay of epifluorescence and DIC images obtained on paraffin section showing microtubules in green (AlexaFluor 488) and cell nuclei in blue (DAPI). Image depicts hindgut epithelium in the posterior part of the papillate region **F** electron micrograph of equivalent area as denoted by frame F in image C. Arrow points to a large bundle of microtubules near the lateral plasma membrane which is indicated by arrowhead **G** electron micrograph of equivalent area as denoted by frame G in image D. Arrowhead points to septate junctions. Arrow points to cross-sectioned microtubules. Abbreviations: CUT – cuticle; HE – hindgut epithelium; HL – hindgut lumen; M – muscles; T – typhlosole; TC – typhlosole channels.

In postmarsupial mancae an intense immunofluorescent labelling of microtubules is visible in epidermal cells, presumably tenocytes, the ventral nerve cord and the hindgut epithelium. The labelling does not differ considerably between postmarsupial mancae at the time of the release from the marsupium and postmarsupial mancae one week after the release from the marsupium. In the anterior chamber all hindgut cells display strong fluorescent labelling (Fig. 9A, B). As in earlier stages the most extensive fluorescent labelling is present in the hindgut epithelium at the transition between the anterior chamber and the papillate region (Fig. 9C). In postmarsupial mancae the typhlosole in this hindgut section becomes anvil shaped and contains particularly large bundles of microtubules. In the anterior part of the papillate region fine bundles of microtubules are distributed throughout the cytosol of hindgut cells (Fig. 9D). In the most posterior part of the papillate region the microtubules are concentrated in the apical part of hindgut cells (Fig. 9E). At the ultrastructural level, the apico-basal microtubule bundles become extensive in postmarsupial mancae and are concentrated in the lateral cytosol near the lateral plasma membranes in the anterior chamber (Fig. 9F). In the papillate region of postmarsupial mancae we observed cross-sectioned microtubules in the vicinity of septate junctions which are not apparent in the earlier stages (Fig. 9G).

### ﻿Typhlosole morphogenesis

**Figure 10.**
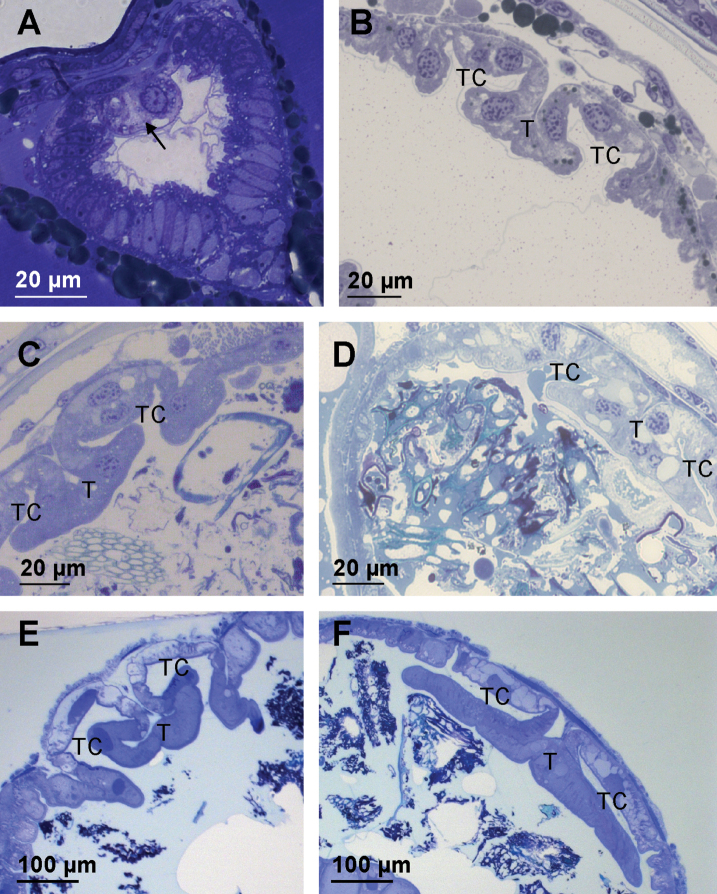
Typhlosole morphogenesis in the hindgut of *P.scaber***A** typhlosole primordium (arrow) in hindgut of late embryo of stage S18 **B** typhlosole in the anterior chamber of marsupial manca **C** typhlosole in the anterior part of the anterior chamber in postmarsupial manca **D** typhlosole in the posterior part of the anterior chamber near the transition to the papillate region in postmarsupial manca **E** typhlosole in the anterior part of the anterior chamber in adult animal **F** typhlosole in the posterior part of the anterior chamber near the transition to the papillate region in adult animal. Abbreviations: T – typhlosole; TC – typhlosole channels.

We have also investigated the morphogenesis of the typhlosole since it is a major remodeling process of the hindgut epithelium and prominent microtubule bundles are present in typhlosole cells. In late embryos a primordium of the typhlosole is established and appears in cross-section as two large dorsal cells bulged into the hindgut lumen (Fig. 10A). Later in marsupial mancae the dorsal hindgut cells already form distinct folds of the typhlosole and two typhlosole channels (Fig. 10B). The morphology of the typhlosole in marsupial mancae is similar along the entire length of the alimentary canal. In postmarsupial mancae the typhlosole at the transition between the anterior chamber and the papillate region widens and becomes anvil shaped (Fig. 10C, D) as in adults (Fig. 10E, F).

## ﻿Discussion

### ﻿Architecture of microtubule bundles in the hindgut cells of adult *P.scaber*

The microtubules are the stiffest of the three cytoskeletal filaments and form almost linear tracks that primarily serve as pathways for the directed intracellular transport (Fletcher and Mullins 2010) and in differentiated cells the microtubules are also important for the maintenance of cell shape (Gelfand and Bershadsky 1991). In combination with other cytoskeletal components, the microtubules can bear considerable compressive forces (Brangwynne et al. 2006), which is particularly important in the regulation of cell height of epithelial cells (Burnside 1973; Picone et al. 2010). Hindgut cells of isopods contain extremely abundant microtubule bundles as reported in *P.scaber* (Bogataj et al. 2018), *O.asellus* (Witkus et al. 1969), *A.vulgare* (Vernon et al. 1974) and two species of marine isopods *D.bidentata* (Holdich and Ratcliffe 1970) and *C.carinata* (Wägele et al. 1981), while microtubule arrangement in the cells of morphologically and functionally distinct hindgut regions has not been studied before.

**Figure 11.**
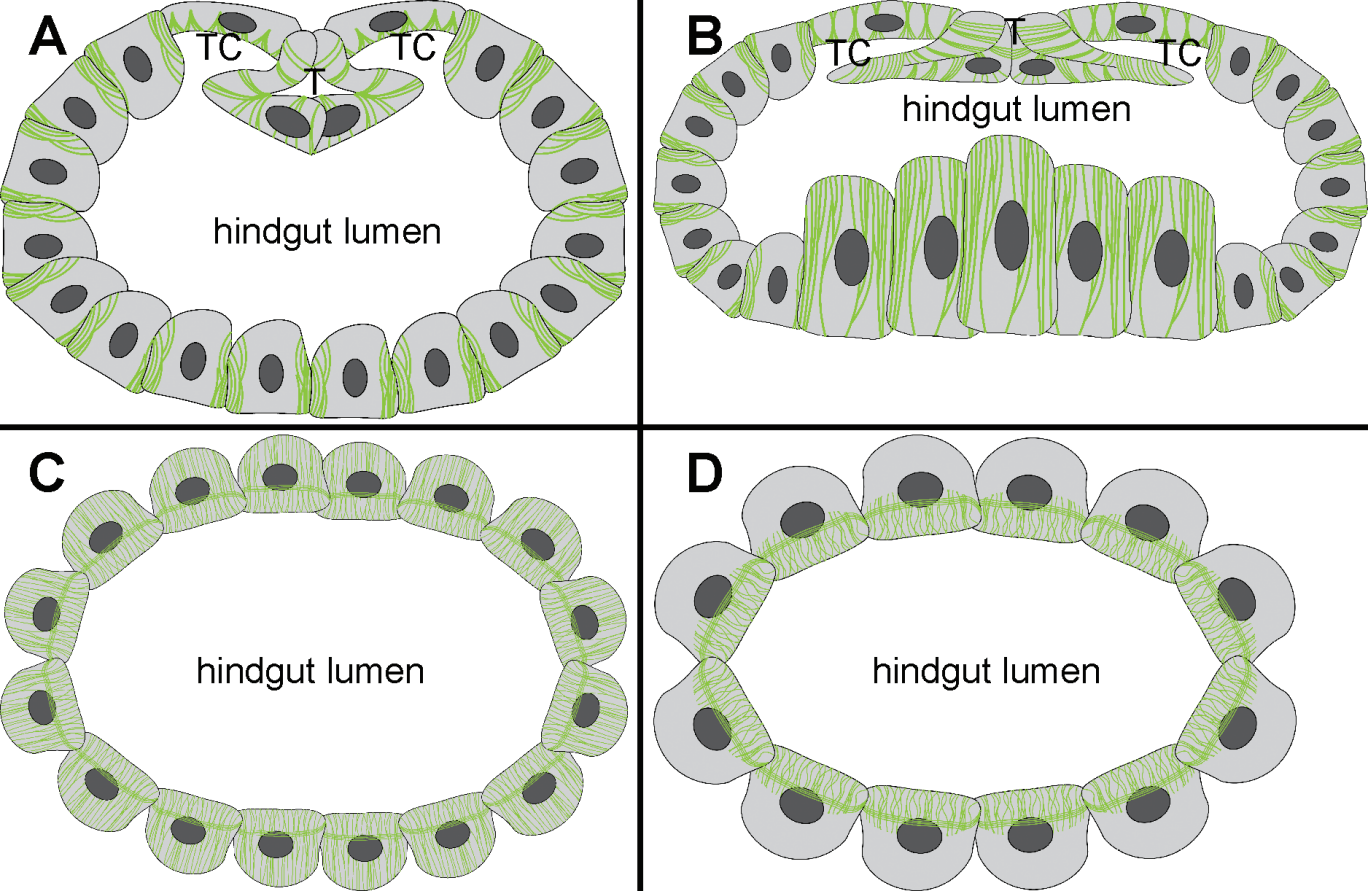
Illustration of microtubule architecture in hindgut cells from different regions of adult *P.scaber* hindgut **A** anterior chamber **B** posterior part of the anterior chamber at the transition to the papillate region **C** papillate region **D** sphincter and rectum.

We show here that the cells in all hindgut regions of adult *P.scaber* contain abundant microtubules which organize into extensive apico-basally oriented bundles. Our results demonstrate that the spatial arrangement of these apico-basal bundles varies considerably in cells from distinct parts of the hindgut that differ markedly in their shape as summarized in the schematic representation (Fig. 11). In the anterior chamber the cells that form the ventral and lateral walls of the hindgut contain relatively thick microtubule bundles concentrated in the vicinity of lateral plasma membranes that are highly interdigitated between the neighboring cells (Fig. 11A). Similar association of microtubule bundles with lateral plasma membranes have been reported in hindgut cells of other isopods (Witkus et al. 1969; Holdich and Ratcliffe 1970; Vernon et al. 1974). In cells that form the typhlosole a specific microtubule arrangement is evident. Long transcellular apico-basal bundles of microtubules are oriented perpendicular to the apical cell surface and distributed along the highly curved outline of typhlosole cells (Fig. 11A). The wide cells around the typhlosole channels contain relatively short densely stacked microtubule bundles in the apical cytosol that ramify in the apical direction (Fig. 11A). In the posterior part of the anterior chamber at the transition to the papillate region the microtubule bundles are the most extensive. The largest microtubule bundles were observed in anvil shaped typhlosole and cells around the typhlosole channels in this transition region as well as in high dome shaped ventral cells (Fig. 11B). In the papillate region all hindgut cells contain fine apico-basal microtubule bundles which appear thinner than in the anterior chamber (Fig. 11C). The microtubule bundles in the papillate region apically protrude into the thin cytoplasmic projections between the infoldings of apical labyrinth and are closely associated with numerous mitochondria. The presence of microtubules in the cytoplasmic projections between the apical infoldings and the association with mitochondria have been reported also in *O.asellus* and *A.vulgare* (Witkus et al. 1969; Vernon et al. 1974). In the papillate region numerous cross-sectioned microtubules near the septate junctions are evident, which likely form a subapical circumferential ring of microtubules perpendicular to the apico-basal ones, but further 3D characterization is needed to verify this assumption. In the sphincter region and rectum, the apico basal microtubule bundles are concentrated in the apical cytosol (Fig. 11D).

In the anterior chamber the most prominent bundles of microtubules are observed in parts of the hindgut epithelium that are associated with extensive visceral muscles. One example are the typhlosole cells, where distinct muscles insert into the typhlosole fold from the basal side (Hames and Hopkin 1989). Another example is the posterior part of the anterior chamber at the transition to the papillate region where extensive network of circular and longitudinal muscles surrounds the hindgut (Vernon et al. 1974; Palackal et al. 1984; Hames and Hopkin 1989). Together with other cytoskeletal elements the microtubules can resist mechanical forces acting on cells and are thus important for the maintenance of cell shape (Wang et al. 2001; Brangwynne et al. 2006; Picone et al. 2010; Sato et al. 2013; Kubitschke et al. 2017). The microtubules are particularly abundant in the tenocytes of arthropods (Nakazawa et al. 1992; Tucker et al. 2004; Criel et al. 2005; Žnidaršič et al. 2012) and cochlear supporting cells of mammals (Tucker et al. 1993; Henderson et al. 1995), which are, similar as the hindgut cells in the anterior chamber of terrestrial isopods, under constant mechanical stress. The extensive microtubule bundles in the typhlosole cells and cells around the typhlosole channels might be involved in the maintenance of the very complex shape of these cells that form the typhlosole fold. This together with the contractions of muscles that insert into the typhlosole fold enables the opening and closing of the typhlosole channels, which is important for the transport of partly digested liquid food during the feeding cycle of terrestrial isopods (Hames and Hopkin 1989). In cells of the lateral and ventral hindgut wall and particularly in large ventral cells in the posterior part of the anterior chamber the apico-basal bundles of microtubules are likely important for the support of the apically dome shaped cells. Accordingly, the hindgut cells can maintain their shape during the peristaltic contractions of extensive musculature surrounding the hindgut, which squeezes the partly digested liquid food into the typhlosole channels and then pushes it anteriorly into the stomach for the filtration and transport into the digestive glands where the absorption takes place (Hames and Hopkin 1989).

In the papillate region the bundles of microtubules protrude deep into the cytoplasmic projections between the infoldings of the apical plasma membrane. In the more posterior sphincter region and rectum the microtubule bundles are concentrated in the apical cytosol where the deep infoldings of apical labyrinth are present in these cells. Thus, the microtubule bundles in the papillate region, sphincter region and rectum could play a role in the stabilization of the deep infoldings of the apical plasma membrane labyrinth, which is important for the function of the papillate region in ion and water transport (Vernon et al. 1974; Coruzzi et al. 1982; Palackal et al. 1984). It is known from studies in different organisms that microtubules with associated molecular motors can deform membranes and are thus important for the positioning and shaping of different cell membranes (Stephens 2012). In cultured mammalian cells it has been shown that the microtubules together with the associated molecular motors also drive the formation of tubular invaginations in the plasma membrane during clathrin independent endocytosis (Day et al. 2015). Microtubules in hindgut cells in the papillate region are in close contact with numerous mitochondria present in the apical cytosol of these cells, which are also aligned in the same apico-basal direction as the microtubules. This is consistent with the role of microtubules in the transport, positioning and shaping of various cell organelles (Tolić-Nørrelykke 2008; Stephens 2012) including the mitochondria (Fujita et al. 2007; Fernández Casafuz et al. 2023).

### ﻿Reorganization of microtubules in relation to hindgut morphogenesis in late embryonic and early postembryonic development

We have characterized the arrangement of microtubules in the hindgut of late-embryos and postembryonic developmental stages and thus provided new data of microtubule arrangement during hindgut morphogenesis in crustaceans. We have observed that the microtubules in the hindgut cells of *P.scaber* organize into distinct apico-basal bundles relatively late during development. In late embryos in stage S18 distinct apico-basal bundles of microtubules are present only in the most posterior part of the hindgut epithelium, in the rectum near the anal opening. Distinct apico-basal bundles of microtubules are evident in the cells of all hindgut regions in the early postembryonic stages of marsupial mancae. The microtubule bundles are gradually increased in length and thickness in postmarsupial mancae. One of the crucial aspects associated with epithelial differentiation and morphogenesis is the rearrangement of the cytoskeletal system in combination with the remodeling of cell junctions. Particularly well understood is the involvement of the actin-myosin cytoskeleton and its contractility in epithelial cell shape change and morphogenetic processes (Lecuit and Lenne 2007). More recent studies have shown that the microtubules also play an important role in cell shape changes and tissue remodeling (Matis 2020), and the involvement of microtubular cytoskeleton in the morphogenesis of invertebrate epithelia is currently an active research topic (Röper 2020). Recent research in *Drosophilamelanogaster* Meigen, 1830 has elucidated that the reorganization of microtubules plays important roles in remodeling of epithelial sheets during morphogenesis. The microtubules promote or directly cause various cell shape changes needed for epithelial folding (Takeda et al. 2018; Ko et al. 2019).

Our data on morphogenesis show that the formation of typhlosole encompass drastic cell shape changes. In late embryos when only a primordium of the typhlosole is present in the anterior hindgut just thin microtubule bundles or possibly single microtubules are present in the cytosol of hindgut cells. Later in marsupial mancae apico-basal microtubule bundles become apparent in dorsal cells that already form distinct folds of the typhlosole and two typhlosole channels. In postmarsupial mancae the apico-basal bundles of microtubules are further expanded and elaborated when the posterior section of the typhlosole widens and becomes anvil shaped. Our results show that the reorganization of microtubules closely coincides with shape changes of typhlosole cells.

## ﻿Conclusions

All cells in adult *P.scaber* hindgut contain abundant apico-basally oriented microtubules. The architecture of these microtubule bundles varies considerably in different parts of the hindgut epithelium along the anterior-posterior axis. In the anterior chamber the thick microtubule bundles are probably involved in the maintenance of cell shape and resistance to the compressive mechanical forces during peristaltic contractions of the hindgut muscles. Specific arrangement of microtubule bundles is evident in the morphologically complex typhlosole fold. In the papillate region the microtubule bundles protrude into the cytoplasmic projections between the deep apical plasma membrane infoldings and might be involved in the stabilization of the apical plasma membrane infoldings and serve in the transport and tethering of mitochondria. During hindgut morphogenesis the apico-basal microtubule bundles are established relatively late, mainly during early postembryonic development. Morphogenesis of the typhlosole is characterized by coinciding changes in cell shape and microtubule arrangement.

## ﻿Additional information

### Author ORCIDs

Polona Mrak https://orcid.org/0000-0003-3642-8191

Nada Žnidaršič https://orcid.org/0000-0002-0164-9050

### Data availability

All of the data that support the findings of this study are available in the main text.
